# Examining the mediating role of resources in the temporal relationship between proactive burnout prevention and burnout

**DOI:** 10.1186/s12889-021-10670-7

**Published:** 2021-03-26

**Authors:** Madelon C. B. Otto, Joris Van Ruysseveldt, Nicole Hoefsmit, Karen Van Dam

**Affiliations:** Faculty of Psychology, Department of Work & Organizational Psychology, Open University, Valkenburgerweg 177, 6419 AT Heerlen, The Netherlands

**Keywords:** Proactive behaviors, Burnout, Prevention, Resources, Mediation

## Abstract

**Background:**

Employees who engage in proactive burnout prevention can prevent burnout by changing aspects of the work, home, and personal domain. However, these proactive behaviors may be impeded by high initial levels of burnout. Based on the conservation of resources theory and the dual-pathway proactivity model, resources were expected to play a vital role in the relationship between proactive burnout prevention and burnout through two distinct processes: a resource-generation process in which proactive burnout prevention negatively affects burnout through an increase in resources, and a resource-depletion process in which proactive burnout prevention is hindered because high initial levels of burnout negatively affected resources.

**Methods:**

A two-wave longitudinal panel design was used in which 617 employees, mainly employed in government agencies, healthcare and education, were asked to complete an online survey twice with an interval of 1 month.

**Results:**

Results of structural equation modelling showed clear evidence for the resource-generation process in the work, home, and personal domain, and only limited evidence for the resource-depletion process. Solely in the personal domain a small negative indirect effect of burnout on proactive burnout prevention through personal resources was found.

**Conclusions:**

The findings of this study confirm that employees can proactively prevent burnout by investing in resources, yet proactive actions should be taken before increased burnout-complaints impede employees to do so. This study contributes to scientific knowledge on proactive behaviors and burnout prevention by investigating the mechanism underlying the temporal relationship between proactive burnout prevention and burnout. An important practical implication of this study is that it highlights that more attention should be given to employees’ self-initiated actions to prevent burnout, as proactive burnout prevention can effectively reduce levels of burnout.

**Supplementary Information:**

The online version contains supplementary material available at 10.1186/s12889-021-10670-7.

Burnout has severe negative consequences for individuals’ physical health and psychological wellbeing [1] as well as organizational outcomes [2], which underscores the importance of burnout prevention. Burnout is an occupational phenomenon that occupational stakeholders and policymakers (i.e., employers, health and safety management, occupational physicians) need to address [3]. Since burnout develops in an interplay between employees and their environment [2], both employers and employees can intervene to prevent burnout. While employer-initiated burnout prevention programs have been studied extensively (see for example, [4, 5], our understanding of how employees themselves can intervene to prevent burnout needs to be enhanced [6].

Recently, Otto et al. [7] have conducted a qualitative study to explore employees’ proactive actions aimed at changing themselves or their environment to prevent burnout. Findings of this study indicate that employees take proactive actions in the work, home, and personal domain to prevent burnout (i.e., proactive burnout prevention [7];). These findings are in line with previous research results showing that burnout is influenced by factors beyond the workplace (e.g., [8, 9]), suggesting that non-work factors need to be included when studying burnout prevention.

Positive relationships between proactive employee behaviors (e.g., taking charge, innovative work behavior) and work-related outcomes (e.g. organizational effectiveness, work performance) are well established [10]. However, temporal associations between proactive behaviors and employee wellbeing, as well as the potential mechanism through which these impact each other have received less research attention [11, 12]. Previous research has shown that proactive burnout prevention has a negative effect on burnout 3, 6, and 9 weeks later, and that initial high levels of burnout may hinder or frustrate proactive burnout prevention [13], indicating the need to better understand the mechanisms underlying this relationship.

According to the conservation of resources (COR) theory [14, 15] resources play a central role in the development of burnout. COR theory posits that individuals strive to obtain and protect valued resources and when these are threatened or lost, respond by investing resources to maintain or restore resources. Drawing on COR theory [14], Cangiano and Parker [11] propose a model of the effect of proactivity on wellbeing, which suggests a dual-pathway via which proactive behaviors affect employees’ wellbeing; positively through a resource-generation pathway, and negatively through a strain pathway [16]. As previous research indicated that proactive burnout prevention has a positive wellbeing outcome (i.e., results in reduced levels of burnout) [13], this could be the result of the resource-generation pathway suggested by Cangiano and Parker’s [11] model. However, at the same time, COR theory predicts that proactive behaviors consume resources, and burned-out employees who experience resource loss, may not have the mental strength or energy to engage in proactive burnout prevention, making them vulnerable to further resource loss and ill health following a resource-depletion process [17, 18].

The goal of this two-wave longitudinal study was to examine the mediating role of resources in the temporal relationship between proactive burnout prevention behaviors and burnout. In line with Cangiano and Parker’s [11] resource-generation pathway a negative lagged indirect effect of proactive burnout prevention on burnout through resources was expected. In addition, a negative reversed indirect effect of burnout on proactive burnout prevention through resources was expected, consistent with the resource-depletion process of COR theory [14].

This study contributes to literature in several ways. First, research into the mechanism underlying the temporal relationship between proactive behaviors and wellbeing outcomes is limited [12]. Therefore, this study enhances scientific knowledge by investigating the mediating role of resources in the temporal relationship between proactive burnout prevention and burnout. Second, Cangiano and Parker’s [11] proposed model of the effect of proactivity on wellbeing was used as one of the theoretical frameworks to examine the mediating effect of resources in this relationship. As far as we know, this model has not been extensively empirically tested, nor has it been linked to proactive burnout prevention. Third, this study not only investigated lagged, but also reversed and combined relationships between proactive behaviors, resources, and burnout. Most of the existing studies only examined lagged effects and/or used cross-sectional designs [12], which inhibits inference regarding causality and reciprocity. Fourth, this study applied an integrative approach to proactive behaviors and burnout prevention, including factors both within and beyond the work environment. To the best of our knowledge, this study is the first to examine the mediating role of resources in the relationship between proactive behaviors and burnout in the work, home, and personal domain.

## Proactive burnout prevention

The definition and assessment of burnout have been under discussion (e.g., [19, 20]). Up until now the Maslach Burnout Inventory (MBI) has been the most used questionnaire to define and measure burnout [19]. However, since the MBI appears to suffer from conceptual, technical and practical imperfections [19, 20], the development of a new tool for assessing burnout was considered necessary [19]. Therefore, Schaufeli, De Witte, and Desart [19, 20] have recently developed the burnout assessment tool (BAT), in which the problems with the MBI were addressed. Although first studies with the BAT show promising results [19, 21], more research is needed to validate this tool and address possible limitations. Notwithstanding the possible shortcomings of the BAT, the burnout assessment tool and its definition of burnout were used in this study, to concur with the latest research developments regarding burnout.

Based on the BAT, burnout is defined as a work-related condition that is characterized by exhaustion, mental distancing, and impairment to regulate emotional and cognitive processes [19, 20]. These four core components can be accompanied by secondary symptoms such as depression and non-specific psychological and psychosomatic distress [19, 20]. Amongst the most influential models for studying job burnout are the job demand-control (JD-C) [22] model and the COR theory [14, 15]. This research draws upon COR theory, because of its ability to base a wider range of hypotheses on than those provided by theories that focus on a single resource [15]. According to COR theory [14], burnout is the result of a resource depletion process caused by on-going exposure to stressors. Resources are those entities valued by individuals that serve as means to achieve goals and include personal (e.g., self-efficacy), social (e.g., co-worker social support), and condition (e.g., autonomy) resources (e.g., [15]). Psychological stress occurs when there is a perceived threat of resource loss, when resources are actually lost, or an anticipated regain of resources is not obtained after an investment in resources [14]. Employees who experience limited resources are more at risk to resource loss, as an initial loss begets future resources loss, leading to spirals of loss which may ultimately leave the employee burned out [17]. To prevent burnout, employees could therefore take initiative to avoid resource depletion, by proactively attempting to increase or maintain resources.

Proactive behaviors are described as self-initiated, change-oriented and future-focused actions aimed at an improved outcome [23]. Over the past years, various proactive concepts, such as voice (speaking up in a constructive manner with an intent to improve rather than merely criticize) and taking charge (voluntary, change-oriented behavior aimed at the improvement of work processes) have been developed and examined in different domains, showing that these behaviors can be beneficial to organizational and individual effectiveness (e.g., [24]). Findings of an exploratory qualitative study [7] indicate that participants who were confronted with high demands in the workplace and/or demanding situations at home (e.g., stressful life events, taking care of elderly family member), reported to take self-initiated actions in the work, home, and personal domain, to protect or (re) gain resources to prevent burnout (i.e., proactive burnout prevention). These study results are consistent with conceptual research [25] and empirical studies [26] suggesting that stressors can prompt proactive behaviors aimed at restoring the imbalance between a current and desired situation to improve wellbeing [23]. To illustrate proactive burnout prevention in the work domain, employees can ask their coworkers for help or advice, if necessary. Extensive research has shown that social support from coworkers is associated with reduced levels of burnout (e.g., [27, 28]), indicating that proactively asking coworkers for help or advice may be effective to prevent burnout. Similarly, in the home domain, employees can ask their family or friends for help or advice. Previous study results showed that social support from family or friends was also related to lower levels of burnout [28, 29], suggesting that proactively seeking social support from family or friends may also be effective to prevent burnout. An example of proactive burnout prevention in the personal domain is improving/maintaining physical health. As previous studies have shown negative relationships between physical health and burnout [30], proactively maintaining or improving one’s physical health is assumed to prevent burnout.

Findings of the aforementioned exploratory study [7] suggested that the participants who did not indicate to be motivated to take proactive action to prevent burnout, appeared to deal with high demands by using non-proactive strategies, such as psychological disengagement and/or alcohol disengagement (e.g., practicing hobbies, drinking alcohol) [31]. These strategies seemed more focused on reactively trying to relief mental and physical strain symptoms on a daily basis, than on proactively addressing the causes of the high demands to avoid burnout. However, it is questionable whether repeatedly attempting to regain lost resources is a successful approach in the long run, as previous research has shown that employees who are exposed to prolonged high job demands may not recover sufficiently from work and suffer from increased levels of burnout [32]. It seems therefore recommendable to take a proactive approach to prevent burnout.

In a subsequent, four-wave panel study, Otto et al. [13] found that proactive burnout prevention negatively affected burnout 3, 6, and 9 weeks later, and conversely, burnout negatively affected proactive burnout prevention 3, 6 and 9 weeks later. These findings indicate that whereas employees who engage in proactive burnout prevention are more likely to prevent burnout, this behavior could be hindered of frustrated by initial high levels of burnout. These results are in line with COR theory [14], which predicts that employees who proactively invest resources to maintain or protect resources may prevent burnout, whereas burned out employees may lack the resources to take proactive action. Based on the findings of Otto et al. [7, 13] this study included the following proactive burnout prevention behaviors in the work home, and personal domain (see Table 1).

**Table 1:** Proactive Burnout Prevention: Domains and Proactive Actions

Domain	Proactive action
**Work**	Increasing/maintaining job control
	Increasing/maintaining supervisor social support
	Increasing/maintaining coworker social support
	Seeking/performing tasks that energize
**Home**	Increasing/maintaining home autonomy
	Increasing/maintaining home social support
	Reducing work-home conflict
**Personal**	Improving/maintaining physical health
	Improving/maintaining psychological wellbeing
	Engaging in relaxing activities

Two proactive burnout prevention behaviors specified in previous research [7] (feedback seeking and reducing hindering job demands) were not included in this study, as the relationship between these proactive burnout prevention behaviors and burnout did not show unequivocal results [13, 33].

## The mediating role of resources

Research into how proactive behaviors affect wellbeing outcomes is scarce [12]. However, knowledge of the potential mechanisms underlying the relationship between proactive burnout prevention and burnout is important, to understand how and why employees differ in their engagement in these behaviors. COR theory [14] and previous study findings [7] suggest that resources play a central role in the relationship between proactive burnout prevention and burnout. Based on COR theory, Cangiano and Parker [11] propose a ‘model of the effect of proactivity on mental health and wellbeing’ consisting of two potential pathways through which proactive behaviors can impact wellbeing outcomes; positive through a resource-generation pathway, where proactivity can for instance boost self-efficacy and increase resources, which in turn can lead to positive wellbeing outcomes; and negative through a strain pathway, where proactivity may possibly create role overload and drains resources, which can lead to negative wellbeing outcomes. Cangiano and Parker [11] posit that whether the wellbeing outcomes of proactive behaviors are positive or negative, is influenced by various contextual (e.g., social support) and individual (e.g., motivation, self-efficacy) factors.

Previous research has shown that proactive burnout prevention can positively affect wellbeing (i.e., the prevention of burnout; [13]). Therefore, it is expected that proactive burnout prevention will follow the resource-generation process as proposed by Cangiano and Parker’s [11], such that employees who engage in these behaviors are expected to prevent burnout out by increasing resources. However, as predicted by COR theory [14], employees who experience high initial levels of burnout may perceive a threat to resources or may have actually lost resources, and are therefore expected to engage less in proactive burnout prevention as a result of reduced resources. Too high initial levels of burnout may therefore hinder proactive burnout prevention following a resource-depletion process. Since burnout develops gradually, proactive actions to prevent burnout should be taken in a timely manner, before resources are lost and the resource pool becomes too depleted to be able to invest resources to maintain or regain resources.

Few studies have used Cangiano and Parker’s [11] propositions to empirically examine the role of resources in the relationship between proactive behaviors and wellbeing. Results of a daily survey study by Cangiano et al. [16] showed that employees’ daily proactive work behavior was positively related to day-levels wellbeing outcomes (i.e., vitality) through personal resources (i.e., perceived competence). However, daily proactive behavior at work undermined end-of-the-work day wellbeing (i.e., detachment) through anxiety, in particular for employees who perceived their supervisor as highly punitive [16].

Results of Zacher et al. ‘s [12] study into the dynamic effects of personal initiative (a broad form of proactive work behavior to overcome barriers and achieve goals; [25]) on engagement and exhaustion, were partially consistent with those of the dual-pathway model [11]. Findings indicated that although high initial levels of proactive behavior (i.e., personal initiative) may expand resources and be beneficial for employees’ wellbeing, an increase in proactive behaviors can have negative consequences, through an inflated expenditure of personal resources (i.e., positive mood). Zacher et al. [12] also examined a reverse causal model; yet this yielded no significant indirect effects.

The present study examined the mediating role of resources in the lagged and reversed relationships between proactive burnout prevention and burnout. The general assumption about the mediating role of resources in the proactive burnout prevention – burnout relationship were applied to three specific domains: work, home and personal. The proposed relationships were analyzed in each domain separately, to obtain a clear and straightforward insight into the underlying mediating mechanism while excluding entangling cross domain interferences. Based on COR theory [14], Cangiano and Parker’s [11] dual-pathway proactivity model and previous research results showing a negative reciprocal relationship between proactive burnout prevention and burnout [13] the following hypotheses were formulated regarding expected relationships in the work, home, and personal domain:

- *Hypothesis 1:* Resources mediate the negative relationship between proactive burnout prevention and burnout, such that proactive burnout prevention has a positive effect on resources, which in turn are negatively related to burnout.
- *Hypothesis 2:* Resources mediate the negative relationship between burnout and proactive burnout prevention, such that burnout has a negative effect on resources, which in turn are positively related to proactive burnout prevention.

## Method

### Study design and participants

This study used a two-wave longitudinal panel design. Participants were recruited through several channels in the Netherlands. Employees of several organizations in different industries were asked by their employer to participate voluntarily in this study. In addition, participants were recruited through the personal network of an intern. Participants had to meet the following inclusion criteria: employees who are 18 years or older and are not on long-term sick leave (6 weeks or longer) at the time of the study. Employees of the participating organizations were informed about the study via an email message or a message on intranet containing a link to the online survey. After clicking on the link, the participants first received detailed information on the study goals and procedure, a notification that participation was voluntary and could be withdrawn at any time during data collection, and information on the storage of data (in line with General Data Protection Regulation [34]). Next, participants were asked for their informed consent, before they could actually start answering questions. Participants were asked to fill out the survey on two measurement occasions with an interval of approximately 1 month. As an incentive, participants were offered to receive their personal risk-profile put together by the researchers, after they completed the survey on both measurement occasions. The study was conducted in accordance with the Declaration of Helsinki [35], and the protocol was approved by an internal academic ethical committee (registration number: U2019/02040/HVM).

Data collection took place from June 2019 to April 2020. At T1 the survey was fully completed by 1088 participants. The exact response rate could not be established, since it was unknown how many people were approached through the personal network of the intern and thus had access to this research. At T2 the survey was completed by 617 participants who also completed the survey at T1 (dropout rate 43%). This final sample was included in the analyses; 56.4% were female, the average age was 44.0 years (*SD* = 11.3), and 57.5% had higher vocational education or a university degree. Participants worked on average 34.7 h per week (*SD* = 7.5) and were mainly employed in three sectors: government (47.3%), healthcare (35.2%), and education (8.8%). Analysis testing for systematic dropout from T1 to T2 revealed no significant differences in terms of gender, education level, average working hours, and industry, but did show a significant difference in age between participants who only completed the survey at T1 and the participants who completed the survey on both occasions. The average age of those who responded on both occasions (*N* = 617) was higher (*M* = 44.0, *SD* = 11.3), than of those who only completed the survey at T1 (*N* = 471; *M* = 41.3, *SD* = 11.4, *t*(1086) = − 2.69, *p* < .001). With regard to the study variables at T1, the dropouts reported a slightly lower level of job resources (*M* = 2.97, *SD* = .43) than the participants who completed the survey on both occasions (*M* = 3.03, *SD* = .45, *t*(1086) = − 2.11, *p* = .035). In addition, the dropouts also scored lower on the proactive burnout prevention behaviors: engaging in relaxing activities (*M* = 3.87, *SD* = .63) and improving/maintaining physical health (*M* = 2.97, *SD* = .43) compared with the participants who completed the survey on T1 and T2 (respectively, *M* = 3.90, *SD* = .64, *t*(1086) = − 2.29, *p* = .022; *M* = 3.03, *SD* = .45, *t(*1086) = − 2.37, *p* = .018), although differences were very small (respectively, Δ*M* = .03 and Δ*M* = .06).

### Measures

#### Proactive burnout prevention in the work domain

Proactive burnout prevention in the work domain was measured at T1 and T2 using 15 items of the Proactive Burnout Prevention Inventory [33] that assessed increasing/maintaining job control, increasing/maintaining supervisor social support, increasing/maintaining coworker social support, and seeking/performing tasks that energize. Sample items are: “I make sure, I am in control of my workload”, and “I actively take on tasks that enable me to develop myself further”. Response categories ranged from 1 (*never*) to 5 *(always*). Cronbach’s alpha was .82 at both T1 and T2.

#### Proactive burnout prevention in the home domain

Proactive burnout prevention in the home domain was measured at T1 and T2 using 9 items of the Proactive Burnout Prevention Inventory [33] that assessed increasing/maintaining home autonomy, increasing/maintaining social support from family/friends, and reducing work-home conflict. Sample items are: “I make sure I can organize my free time myself” and “I make sure I distance myself from work after hours”. Response categories ranged from 1 (*never*) to 5 *(always*). Cronbach’s alpha was .78 at T1 and .79 at T2.

#### Proactive burnout prevention in the personal domain

Proactive burnout prevention in the personal domain was measured at T1 and T2 using 10 items of the Proactive Burnout Prevention Inventory [33] that assessed improving/maintaining physical health, improving/maintaining psychological wellbeing, and engaging in relaxing activities. Sample items are: “I make sure I engage enough in sports”, and “I try to put stressful situations in perspective” Response categories ranged from 1 (*never*) to 5 *(always*). Cronbach’s alpha was .83 at both T1 and T2.

#### Job resources

Autonomy and social support were used to operationalize job and home resources, as research evidence consistently shows that these are protective factors for the development of burnout (e.g., [27, 29]). Job resources were measured at T1 and T2 using the following 9 items: three items that assessed job autonomy and three items that assessed coworker social support from a scale developed by Bakker, Demerouti, and Verbeke [36], based on the questionnaire of experience and evaluation of work (QEEW) by Van Veldhoven and Meijman [37], and three items that mirrored the items to assess coworker social support were used to assess supervisor social support. Example items are: “Do you have control over how your work is carried out?”, “If necessary, can you ask your colleagues for help?”, and “If necessary, can you ask your supervisor for advice?”. Response categories ranged from 1 (*never*) to 4 *(always*). Cronbach’s alpha was .82 at T1 and .81 at T2.

#### Home resources

Home resources were measured at T1 and T2 using the following 6 items: three items assessing home autonomy and three items assessing social support from family/friends [36]. These 6 items mirrored the items used to measure job autonomy and coworker/supervisor social support [8]. Example items are: “Do you have control over how you spend your free time?” and “If necessary, can you ask your family/friends for help?”. Response categories ranged from 1 (*never*) to 4 *(always*). Cronbach’s alpha was .79 at T1 and .84 at T2.

#### Personal resources

Self-efficacy and optimism were used to operationalize personal resources [38]. Personal resources were measured by six items that assessed self-efficacy using the Dutch adaption of the short version of the General Self-Efficacy Scale (GSE-6 [39];) and three items that assessed optimism using the Dutch version of the optimism scale of the shortened Life Orientation Test-Revised (LOT-R [40];). Example items are: “I can always manage to solve difficult problems if I try hard enough” and “I’m always optimistic about my future”. Response categories ranged from 1 (strongly disagree) to 5 *(strongly agree*). Cronbach’s alpha was .81 at T1 and .83 at T2.

#### Burnout

Burnout was measured at both measurement occasions using the 23 item Burnout Assessment Tool [20], covering the four core components extreme exhaustion, emotional and cognitive impairment, and mental distancing. An example item is: “At work, I feel physically exhausted”. Response categories ranged from 1 (*never*) to 5 *(always*). Cronbach’s alpha was .95 at both T1 and T2.

### Analysis

To test the hypotheses, structural equation modelling (SEM) was performed with maximum likelihood estimation using AMOS 25. First, to verify the stability of the measures over time, configural and metric invariance for the latent variables at both measurement occasions was examined. Multi-group confirmatory factor analysis was conducted to test the same measurement model at both occasions [41]. The model specified that proactive burnout prevention items in a specific domain should load on a latent variable proactive burnout prevention in this domain. This approach was applied to all three domains. For resources, the model similarly specified that resources in a specific domain should load on a latent variable resources in this domain. This approach was again applied to all three domains. Also, for burnout, the model similarly specified that the individual items should load on a latent variable burnout.

**Fig. 1:**
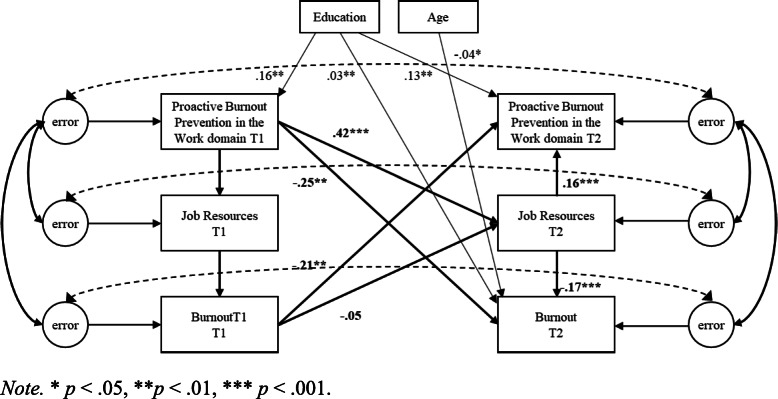
Proactive Burnout Prevention in the Work Domain, Job Resources, and Burnout. *Note.* * *p* < .05, **p < .01, *** *p* < .001

**Fig. 2:**
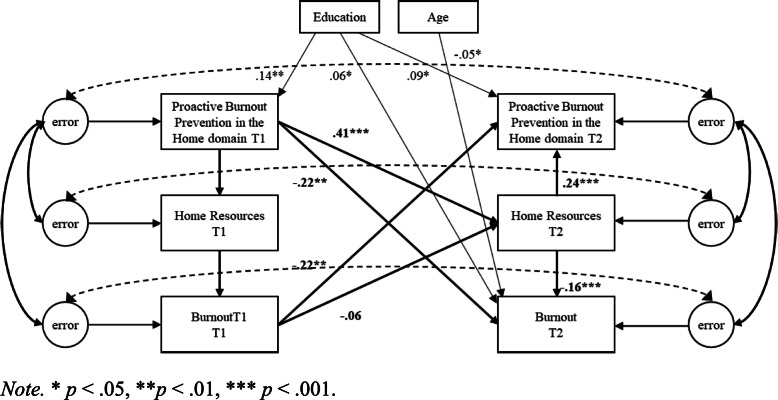
Proactive Burnout Prevention in the Home Domain, Home Resources, and Burnout. *Note.* * *p* < .05, **p < .01, *** *p* < .001

**Fig. 3:**
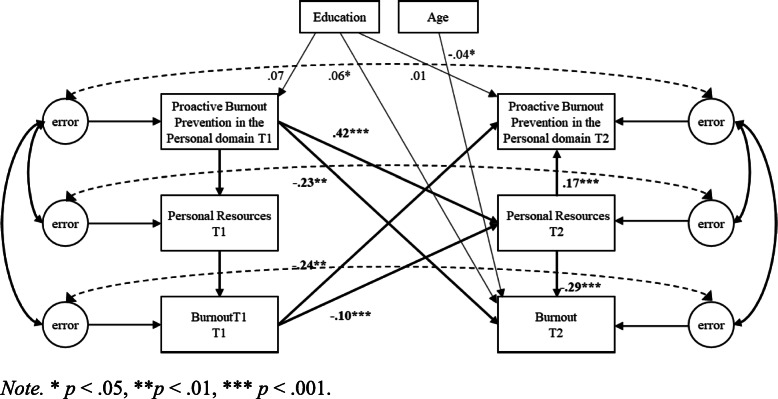
Proactive Burnout Prevention in the Personal Domain, Personal Resources, and Burnout. *Note.* * *p* < .05, **p < .01, *** *p* < .001

Second, cross-lagged SEM models using robust standard error maximum likelihood estimation were used to test the hypotheses in the work, home, and personal domain separately. Proactive burnout prevention, resources, and burnout were included in the structural equation model as latent variables [42]. The error terms of the latent factors at T1 and T2 were allowed to covary [43]. Three competing nested models were compared for each of the three domains separately. Model 1 (M1) included cross-lagged structural paths from T1 proactive burnout prevention to T2 resources, and T2 burnout, and from T2 resources to T2 burnout; Model 2 (M2) included cross-lagged structural paths from T1 burnout to T2 proactive burnout prevention and T2 resources, and from T2 resources to T2 proactive burnout prevention; Model 3 (M3) included both aforementioned cross-lagged structural patterns (normal and reversed causation), representing combined effects (see Figs. 1, 2 and 3).

Model fit was assessed using the model chi-square goodness-of-fit with degrees of freedom, Tucker Lewis Index (TLI), comparative fit index (CFI), root mean square of approximation (RMSEA), and the standardized root mean square residual (SRMR) [41, 44]. Values of TLI and CFI higher than .90 are considered acceptable fit, values higher than .95 indicate good fit [41, 44]. RMSEA values below .08 suggest good fit, values ranging from .08 to .10 indicate mediocre fit, and those higher than .10 indicate poor fit [44]. SRMR indicates acceptable fit when it produces a value lower than .10, it can be interpreted as the indicator of good fit when it produces a value lower than .08 [45]. Chi-square difference testing was used to compare the models [46]. A significant improvement in the χ^2^ value indicates a better fit of the model. Bootstrapping analyses were conducted to establish the significance of indirect effects.

## Results

### Descriptive statistics

Table 2 presents means, standard deviations, Cronbach’s alpha’s, and correlations between the study variables. The sociodemographic variables age and education correlated with study variables and were therefore controlled for in the analysis.

**Table 2:** Means, Standard Deviations, Cronbach’s Alpha, and Correlations Between Study Variables

	Variable	M	SD	α	1	2	3	4	5	6	7	8	9	10	11	12	13	14	15	16
1	Age	43.98	11.34		–															
2	Education	8.62	1.62		−.10*	–														
3	PBP_work (T1)	3.62	0.44	.82	−.13**	.16**	–													
4	PBP_home (T1)	3.34	0.53	.78	−.06	.09*	.36**	–												
5	PBP_personal (T1)	3.76	0.50	.83	.07	.06	.41**	.50**	–											
6	Job Resources (T1)	3.03	0.45	.82	−.02	.07	.51**	.16**	.29**	–										
7	Home Resources (T1)	3.10	0.48	.79	.02	.05	.31**	.50**	.48**	.37**	–									
8	Personal Resources (T1)	3.48	0.49	.81	.07	.05	.32**	.24**	.47**	.32**	.33**	–								
9	Burnout (T1)	1.94	0.57	.95	.01	−.00	−.36**	−.30**	−.42**	−.42**	−.33**	−.47**	–							
10	PBP_work (T2)	3.60	0.41	.82	−.18**	.12**	.75**	.36**	.37**	.42**	.30**	.30**	−.33**	–						
11	PBP_home (T2)	3.38	0.52	.79	−.09*	.03	.33**	.75**	.45**	.19**	.45**	.26**	−.32**	.45**	–					
12	PBP_personal (T2)	3.78	0.47	.83	−.08*	.01	.36**	.44**	.81**	.27**	.46**	.49**	−.39**	.38**	.48**	–				
13	Job Resources (T2)	3.04	0.43	.81	−.01	.03	.44**	.18**	.28**	.70**	.32**	.26**	−.35**	.47**	.24**	.32**	–			
14	Home Resources (T2)	3.09	0.51	.84	−.05	.01	.23**	.43**	.44**	.28**	.68**	.31**	−.29**	.31**	.53**	.53**	.36**	–		
15	Personal Resources (T2)	3.52	0.49	.83	.03	.03	.29**	.23**	.46**	.26**	.31**	.75**	−.46**	.37**	.31**	.53**	.35**	.36**	–	
16	Burnout (T2)	1.93	0.56	.95	−.03	.05	−.32**	−.37**	−.37**	−.37**	−.33**	−.45**	.86**	−.33**	−.33**	−.42**	−.37**	−.35**	−.52**	–

*Note. N* = 617. Education = 1 ‘no schooling completed’ to 11 ‘master, PhD, post-doc’

*PBP* Proactive Burnout Prevention

* *p* < .05

**p < .01

### Measurement invariance

Table 3 depicts the results of the measurement invariance tests of the seven study variables (proactive burnout prevention in the work, home, and personal domain, job, home, and personal resource, and burnout). As can be seen from the results, applying the same structure for the study variables at T1 and T2 provided good fit for all study variables (TLI and CFI > .95; RMSEA < .080; SRMR < .080). Constraining the factor loadings did not significantly change the fit, as chi-square difference testing showed no significant results (all *p*-values > .05) for all study variables. Moreover, all items loaded significantly on the latent variables for all study variables (all *p*-values < .01). In sum, the results provided evidence for measurement invariance for all study variables.

**Table 3:** Fit of Measurement Models to Test Measurement Invariance Study Variables

	Model	χ^2^	df	TLI	CFI	RMSEA	SRMR
PBP_w_	Unconstrained	306.21***	136	.97	.98	.032	.042
	Factor loadings constrained	319.20***	150	.97	.98	.030	.046
PBP_H_	Unconstrained	43.14	36	.99	.99	.013	.021
	Factor loadings constrained	69.43	54	.99	.99	.015	.022
PBP_P_	Unconstrained	57.12	64	.99	.99	.014	.024
	Factor loadings constrained	76.53*	55	.99	.99	.018	.027
Job Resources	Unconstrained	113.37***	36	.98	.99	.042	.022
	Factor loadings constrained	121.46***	44	.98	.99	.038	.029
Home Resources	Unconstrained	16.98*	8	.99	.99	.030	.011
	Factor loadings constrained	25.19*	13	.99	.99	.028	.039
Personal Resources	Unconstrained	203.26***	48	.94	.96	.051	.040
	Factor loadings constrained	214.27***	56	.94	.96	.048	.043
Burnout	Unconstrained	622.62***	344	.98	.99	.026	.025
	Factor loadings constrained	638.53***	366	.98	.99	.025	.026

*Note*. *N* = 617

*PBP*_*W*_ Proactive Burnout Prevention in the work domain, *PBP*_*H*_ Proactive Burnout Prevention in the home domain, *PBP*_*P*_ Proactive Burnout Prevention in the personal domain

*p < .05

***p < .001

### Model and hypotheses testing

Model comparison (depicted in Table 4) indicated that in each domain the combined model (M3) exhibited the best level of fit to the data (i.e., work domain: *χ*^*2*^(*df* = 26) *=* 62.74, *p* < .001; TLI = .93; CFI = .98; RMSEA = .093; SRMR = .051, home domain: (*χ*^*2*^(*df* = 26) *=* 37.75, *p* < .001; TLI = .96; CFI = .99; RMSEA = .068; SRMR = .037; personal domain: *χ*^*2*^(*df* = 26) *=* 32.33, *p* < .001; TLI = .98; CFI = .99; RMSEA = .060; SRMR = .039). Moreover, Model 3 showed a significantly better fit to the data compared to the lagged model (M1) (i.e., work domain: Δ*χ*^*2*^(Δ*df* = 2) = 31.70, *p* < .001; home domain: Δ*χ*^*2*^(Δ*df* = 2) = 32.28, *p* < .001; personal domain: Δ*χ*^*2*^(Δ*df* = 2) = 48.29, *p* < .001) and reversed model (M2) (i.e., work domain: Δ*χ*^*2*^(Δ*df* = 2) = 154.32, *p* < .001; (Δ*χ*^*2*^(Δ*df* = 2) = 151.57, *p* < .001; personal domain: (Δ*χ*^*2*^(Δ*df* = 2) = 158.36, *p* < .001). Since in each domain Model 3 fitted the data better than the lagged and reversed models, this model was used to interpret the lagged and reversed effects. The control variables age and education were significant predictors of burnout within all three domains, and significant predictors of proactive burnout prevention in the work and home domain (see Figs. 1, 2 and 3).

**Table 4:** Goodness-of Fit Indices of the Models to Test the Hypotheses

Model	χ^2^	Df	TLI	CFI	RMSEA	SRMR	Comparison	Δ Χ^2^	Δdf
**Work domain**									
M1 PBP_W_T_1_ - > R_J_T_2_ - > BOT_2_	94.44***	24	.91	.96	.106	.084			
M2 BOT_1_ - > R_J_T_2_ - > PBP_W_T_2_	217.06***	24	.78	.96	.167	.139			
M3 Both paths	62.74***	26	.93	.98	.093	.051	M1 vs M3	31.70***	2
							M2 vs M3	154.32***	2
**Home domain**									
M1 PBP_H_T_1_ - > R_H_T_2_ - > BOT_2_	73.03***	24	.93	.97	.093	.082			
M2 BOT_1_ - > R_H_T_2_ - > PBP_T_T_2_	180.32***	24	.82	.92	.151	.126			
M3 Both paths	37.75***	26	.96	.99	.068	.037	M1 vs M3	32.28***	2
							M2 vs M3	151.57***	2
**Personal domain**									
M1 PBP_P_T_1_ - > R_P_T_2_ - > BOT_2_	80.62***	24	.94	.97	.096	.081			
M2 BOT_1_ - > R_P_T_2_ - > PBP_P_T_2_	190.69***	24	.83	.93	.155	.143			
M3 Both paths	32.33***	26	.98	.99	.060	.039	M1 vs M3	48.29***	2
							M2 vs M3	158.36***	2

*Note*. *N* = 617

*PBP*_*W*_ Proactive Burnout Prevention in the work domain, *R*_*J*_ Job Resources, *PBP*_*H*_ Proactive Burnout Prevention in the home domain, *R*_*H*_ Home Resources, *PBP*_*P*_ Proactive Burnout Prevention in the personal domain, *R*_*P*_ Personal Resources, *BO* Burnout

***p < .001

Hypothesis 1 predicted for all three domains separately that resources mediate the negative relationship between proactive burnout prevention and burnout, such that proactive burnout prevention has a positive effect on resources, which in turn are negatively related to burnout. Results supported Hypothesis 1 for the work, home, and personal domain (see Figs. 1, 2 and 3). Proactive burnout prevention in the work domain positively affected job resources 1 month later (β = .42, *p* < .001), which in turn were negatively related to burnout (β = −.17, *p* < .001). Moreover, a significant negative effect of proactive burnout prevention in the work domain on burnout 1 month later was found (β = −.32, BCa CI [−.388, −.241]), and this relationship was mediated by job resources (β = −.09, BCa CI [−.105, −.040]). Proactive burnout prevention in the home domain positively affected home resources 1 month later (β = .41, *p* < .001), which in turn were negatively related to burnout (β = −.16, *p* < .001). Additionally, a significant negative effect of proactive burnout prevention in the home domain on burnout 1 month later was found (β = −.29, BCa CI [−.362, −.201]), and this relationship was mediated by home resources (β = −.06, BCa CI [−.095, −.038]). Proactive burnout prevention in the personal domain positively affected personal resources 1 month later (β = .42, *p* < .001), which in turn were negatively related to burnout (β = −.29, *p* < .001). In addition, a significant negative effect of proactive burnout prevention in the personal domain on burnout 1 month later was found (β = −.36, BCa CI [−.434, −.283]), and this relationship was mediated by personal resources (β = −.13, BCa CI [−.161, −.093]).

Hypothesis 2 predicted for all three domains separately that resources mediate the negative relationship between burnout and proactive burnout prevention, such that burnout has a negative effect on resources, which in turn are positively related to proactive burnout prevention. Results did not support Hypothesis 2 for the work and home domain, whereas they did support Hypothesis 2 for the personal domain, although the indirect effect was small. In the work domain, no significant negative effect of burnout on job resources 1 month later was found (β = −.05, *p* = .129), yet results showed a positive relationship between job resources and proactive burnout prevention in the work domain (β = .16, *p* < .001). Moreover, although results showed a significant negative effect of burnout on proactive burnout prevention in the work domain 1 month later (β = −.22, BCa CI [−.306, −.128]), no mediating effect of job resources on this relationship was found (β = −.01, *p* = .128, BCa CI[−.022, .003]). In the home domain, results did not show a significant negative effect of burnout on home resources 1 month later (β = −.06, *p* = .061), yet results did show a positive relationship between home resources and proactive burnout prevention in the home domain (β = .24, *p* < .001). Although a significant negative effect of burnout on proactive burnout prevention in the home domain 1 month later was found (β = −.23, BCa CI [−.312, −.159]), results showed no mediating effect of home resources on this relationship (β = −.01, *p* = .128, BCa CI [−.032, .001]). In the personal domain, burnout negatively affected personal resources 1 month later (β = −.10, *p* < .001), which in turn positively affected proactive burnout prevention in the personal domain (β = .17, *p* < .001). Additionally, a significant negative effect of burnout on proactive burnout prevention in the personal domain 1 month later was found (β = −.26, BCa CI [−.338, −.175]), and this relationship was mediated by personal resources (β = −.02, BCa CI [−.034, −.006]).

## Discussion

The goal of the present study was to examine the mediating role of resources in the temporal relationship between proactive burnout prevention and burnout. Based on COR theory [14], the dual-pathway model of the effect of proactivity on wellbeing [11], and previous research results [13], a two-wave panel design was used to investigate whether employees who engage in proactive burnout prevention are more likely to prevent burnout through increased levels of resources (i.e., resource-generation process) and whether these behaviors are inhibited as result of reduced levels of resources caused by high initial levels of burnout (i.e., resource-depletion process). These processes were examined in the work, home, and personal domain separately, to gain a clear and forthright insight into the mediating mechanism underlying the proactive burnout prevention – burnout relationship, while excluding intertwining cross domain inferences.

Findings of this study supported the resource-generation process; within all three domains a negative indirect effect of proactive burnout prevention on burnout through resources was found, such that proactive burnout prevention positively affected resources, which in turn were negatively related to burnout. These found lagged effects are consistent with Cangiano and Parker’s [11] notion and empirical study findings [12, 16] that proactive behaviors can positively affect wellbeing following a resource-generation pathway. Specifically, results confirm that proactive burnout prevention can be effective in reducing levels of burnout through their positive effect on resources. This important result indicates that employees themselves can be in charge of building or protecting the resources they value or need to prevent burnout. This study did not find evidence for Cangiano and Parker’s [11] proposed strain pathway, indicating a negative effect of proactive behavior on wellbeing, as proactive burnout prevention positively affected resources, which in turn were related to reduced levels of burnout in all three domains.

In addition to the found negative indirect effect of proactive burnout prevention on burnout through increased resources, results of examining the lagged effects showed that proactive burnout prevention has a direct negative effect on burnout as well. Taking proactive action to prevent burnout may have already influenced employees’ experienced burnout complaints, although these actions may not (yet) have led to an actual increase in resources. Previous research suggests that beneficial effects of proactive behaviors on wellbeing may simply stem from engaging in these behaviors [47, 48]. However, findings also indicate that there may be other mechanisms underlying the temporal relationship between proactive burnout prevention and burnout.

Contrary to expectations, the results of examining the reversed effects of burnout on proactive burnout prevention through resources only showed limited evidence for the hypothesized resource-depletion process. Only in the personal domain a small negative indirect effect of burnout on proactive burnout prevention through personal resources was found. However, consistent with previous research results [13], there was a negative direct effect of burnout on proactive burnout prevention within all three domains. Also, in line with previous research that indicated that individual and contextual factors resources predict proactive behaviors [10], resources were positively related to proactive burnout prevention.

Unexpectedly, findings showed no meaningful negative direct (reversed) effect of burnout on resources, apart from a small negative effect of burnout on personal resources in the personal domain. As such, these findings are largely inconsistent with one of the main corollaries of COR theory that initial resource loss instigates further loss leading to loss cycles [17]. Previous research confirmed that burnout, reflecting a situation of resource loss [14], can induce a loss cycle [49]. Ten Brummelhuis et al. [49] found that initial levels of burnout predicted future burnout through a decrease in job resources and an increase in job demands. Since the initial levels of burnout measured in the study of Ten Brummelhuis et al. [49] appeared on average to be higher than the initial levels of burnout measured in the current study, this may have influenced results. The mean score on burnout at T1 was 1.94, which refers to the response category ‘seldom’. This may have made it difficult or unnecessary to reduce burnout further over time. In fact, higher initial levels of burnout indicate higher vulnerability to further resource loss [17]. More longitudinal research is needed to investigate at what level of burnout complaints it becomes difficult or impossible to engage in proactive burnout prevention.

The findings of examining the reversed effects of burnout on proactive burnout prevention are consistent with previous research by Zacher et al. [12], who also found no negative effects of exhaustion on personal and job resources, and thus no mediating effect of personal and job resources in the relationship between exhaustion and personal initiative. A possible explanation for the differences found in effects of burnout on resources between the study of Ten Brummelhuis et al. [49] on one hand and the present study and the study by Zacher et al. [12] on the other hand, may be the difference in time intervals used in the various studies. The current study used a one-month time interval, Zacher et al. [12] used two, six-month time intervals, and Ten Brummelhuis et al. [49] used a 2 year time interval to examine effects. Burnout develops gradually over time [14] and employees who experience burnout complaints become protective and defensive of their remaining resources to prevent further resource depletion [50]; it may therefore take longer than a few months before the negative effects of burnout on resources become evident.

In sum, examination of the lagged and reversed effects of proactive burnout prevention in the work, home, and personal domain on burnout revealed that, within all three domains, proactive burnout prevention can prevent burnout directly and indirectly through an increase in resources and, conversely, burnout can hinder or frustrate the engagement in these behaviors. Moreover, consistent with previous research [13], the specified combined effects model (including both lagged and reversed effects) showed better fit to the data than the separate lagged and reversed models within all three domains, indicating that proactive burnout prevention and burnout mutually influence each other negatively over time. These findings indicate that proactive burnout prevention within and outside of the workplace is effective in preventing burnout by positively affecting resources, yet employees should take action in a timely manner, before burnout complaints hinder or frustrate them from doing so.

This study was the first to investigate the temporal relationships between proactive behaviors and burnout in the work, home, and personal domain separately. Previous research of potential effects of proactive behaviors on wellbeing are limited [12] and most research has focused on the relationships between proactive behaviors in the work environment and wellbeing. Findings of this study add to knowledge by showing that not only proactive burnout prevention in the work domain is effective in preventing burnout, but proactive burnout prevention in both the home and personal domain contribute to reduced levels of burnout as well. This confirms that to prevent burnout it is important to include factors within and beyond the workplace.

### Limitations and directions for future research

A few methodological limitations regarding the present study should be recognized. First, a novel instrument (burnout assessment tool – BAT) was used to define and assess burnout [19]. Although first studies on the validity and reliability of the BAT show promising results, possible limitations regarding this tool and its definition of burnout still need to be addressed in future research [19, 21]. In addition, in this study the core components of the BAT were assessed (extreme exhaustion, emotional and cognitive impairment, and mental distancing) and not the secondary symptoms (psychological and psychosomatic complaints) [19, 20]. As such, this limits our inferences on the relationship between proactive burnout prevention and burnout, to these four dimensions.

Second, the results were based on self-reports, which are prone to social desirability and common method bias [51]. Although measures were in place to prevent this, such as giving clear instructions and emphasizing that results would be handled confidentially and could not be traced back to individual employees, future studies might also include more objective ratings of employees’ proactive burnout prevention behaviors provided by their supervisors, coworkers, family members and/or friends.

Third, although the sample was heterogenous in terms of gender, age, and education, almost half of the participants were employed in government agencies. This may limit generalizability to employees in other sectors, such as transportation, construction, and production. A certain level of autonomy is required to engage in proactive behavior [10]. Previous research results have found differences in the extent to which employees experience autonomy in different industries [52], suggesting that in some industries it may be more difficult to take proactive actions to prevent burnout, than in others.

Fourth, another possible limitation in terms of the generalizability of the results may be that this study was conducted in the Netherlands and all participants were Dutch. Research has shown that engagement in proactive behaviors in individualist national cultures, such as the Netherlands, is higher than in collectivist national culture [53]. Future studies should include participants from more industries and nationalities to examine whether the findings can be replicated in other sectors and cultures.

In the present study, resources were examined as underlying mechanism in the relationship between proactive behaviors and burnout. Instead of resources, future studies could investigate the mediating role of demands in the temporal relationship between proactive burnout prevention and burnout. As mentioned, Ten Brummelhuis et al. [49] found that burnout induces loss cycles not only through decreased resources, but also through increased job demands. Bakker and Costa [54] suggest that burned-out employees are less able to focus on tasks and make more mistakes due to health problems (e.g., cognitive impairments, insomnia), resulting in an accumulation of job demands. Research on the mediating effect of demands in the relationship between proactive behaviors and burnout is scarce and focused on work-related demands [48]. In addition to job demands, the role of home and personal demands in the relationship between proactive behaviors and burnout could be included in future studies [38].

Future studies could also investigate other factors that may have an impact on the temporal relationship between proactive burnout prevention and burnout. For instance, conceptual and empirical research [11, 55] indicate that the relationship between proactive behaviors and wellbeing outcomes is moderated by type of feedback (positive of negative) and motivation (autonomous vs controlled). Moreover, boundary conditions, such as a supportive social climate and leadership style also seem to affect the use and effect of proactive behaviors [10, 23].

A last suggestion for further investigation is to examine how proactive burnout prevention, as self-initiated action to prevent burnout, relates to employer-initiated interventions to prevent burnout. Meta-analytical review studies of burnout prevention interventions have shown that these programs have small, albeit lasting effects [4, 5]. Moreover, most of these interventions focused on employer-initiated interventions in the workplace and have generally not included factors outside the work environment. The results of the present study indicate that tailored individual strategies, in the form of proactive burnout prevention, may complement top-down interventions to increase the overall effectiveness of burnout prevention interventions.

### Practical implications

An important practical implication of this study is that (more) attention should be given to employees’ self-initiated actions to prevent burnout, as proactive burnout prevention can effectively decrease levels of burnout, provided these behaviors are timely activated. Not only does proactive burnout prevention have a direct impact on reducing levels of burnout, but these behaviors can also boost resources, which in turn positively affect employee wellbeing. Based on the findings of the present study, a self-management intervention could be developed, which creates awareness for proactive burnout prevention and inspires employees to develop these behaviors. This does not imply that employees alone are responsible for burnout prevention. Foremost, employers should be made aware that employees will only engage in proactive behaviors if they feel safe enough and encouraged to do so [10].

## Conclusions

This study contributes to literature by enhancing our understanding of the mechanism underlying the temporal relationship between proactive behaviors and burnout. Findings of this study confirm that employees can proactively prevent burnout by investing in resources. Yet, such proactive burnout prevention may be impeded by high initial levels of burnout. Results indicate that an integrative approach to burnout prevention is recommended, as employees’ proactive actions in the work, home, and personal domain negatively affected burnout. More attention should be given to employees’ self-initiated actions to prevent burnout.

## Supplementary Information

**Additional file 1:.** Means and standard deviations of proactive burnout prevention inventory items (T1).

**Additional file 2:.** BCa CI intervals.

**Additional file 3: Supplementary Material.** Proactive Burnout Prevention Inventory.

## Data Availability

The datasets generated during and/or analyzed during the current study are available from the corresponding author on reasonable request.
