# Supplementary material for: Examining the mediating role of resources in the temporal relationship between proactive burnout prevention and burnout
Source: BMC Public Health. 2021 Mar 26;21:599. doi: 10.1186/s12889-021-10670-7 (PMC8004439; doi:10.1186/s12889-021-10670-7)
Supplement: Supplementary file 2 — Additional file 2:. BCa CI intervals. [file 12889_2021_10670_MOESM2_ESM.docx]

Examining the Mediating Role of Resources in the Temporal Relationship between Proactive Burnout Prevention and Burnout

Madelon C.B. Otto, Joris Van Ruysseveldt, Nicole Hoefsmit, Karen Van Dam, Faculty of Psychology, Department of Work & Organizational Psychology, Open University, Heerlen, The Netherlands.

Author Note

Correspondence concerning this article should be addressed to Madelon C.B. Otto, Faculty of Psychology, Department of Work & Organizational Psychology, Valkenburgerweg 177, 6419 AT, Heerlen, The Netherlands.

E-mail: madelon.otto@ou.nl

**Supplementary Material: BCa CI intervals**

|  | | BCa CI | |
| --- | --- | --- | --- |
|  | | Lower | Upper |
| **Work domain** | |  |  |
|  | PBP_W_T_1_ -> BOT_2_ | -.388 | -.241 |
|  | PBP_W_T_1_ -> R_J_T_2_ -> BOT_2_ | -.105 | -.040 |
|  | BOT_1_ -> PBP_W_T_2_ | -.306 | -.128 |
|  | BOT_1_ -> R_J_T_2_ -> PBP_W_T_2_ | -.022 | .003 |
|  |  |  |  |
| **Home domain** | |  |  |
|  | PBP_H_T_1_ -> BOT_2_ | -.362 | -.201 |
|  | PBP_H_T_1_ -> R_H_T2 ->BOT_2_ | -.095 | -.038 |
|  | BOT_1_ -> PBP_T_T_2_ | -.312 | -.159 |
|  | BOT_1_ -> R_H_T_2_ -> PBP_T_T_2_ | -.032 | .001 |
|  |  |  |  |
| **Personal domain** | |  |  |
|  | PBP_P_T_1_ -> BOT_2_ | -.434 | -.283 |
|  | PBP_P_T_1_ -> R_P_T_2_ -> BOT_2_ | -.161 | -.093 |
|  | BOT_1_ -> PBP_P_T_2_ | -.338 | -.175 |
|  | BOT_1_ -> R_P_T_2_ -> PBP_P_T_2_ | -.034 | -.006 |
|  |  |  |  |
