# Supplementary material for: Examining the mediating role of resources in the temporal relationship between proactive burnout prevention and burnout
Source: BMC Public Health. 2021 Mar 26;21:599. doi: 10.1186/s12889-021-10670-7 (PMC8004439; doi:10.1186/s12889-021-10670-7)
Supplement: Supplementary file 3 — Additional file 3: Supplementary Material. Proactive Burnout Prevention Inventory. [file 12889_2021_10670_MOESM3_ESM.docx]

Examining the Mediating Role of Resources in the Temporal Relationship between Proactive Burnout Prevention and Burnout

Madelon C.B. Otto, Joris Van Ruysseveldt, Nicole Hoefsmit, Karen Van Dam, Faculty of Psychology, Department of Work & Organizational Psychology, Open University, Heerlen, The Netherlands.

Author Note

Correspondence concerning this article should be addressed to Madelon C.B. Otto, Faculty of Psychology, Department of Work & Organizational Psychology, Valkenburgerweg 177, 6419 AT, Heerlen, The Netherlands.

E-mail: madelon.otto@ou.nl

**Supplementary Material: Proactive Burnout Prevention Inventory**

| Proactive burnout prevention in the work domain | |
| --- | --- |
|  | I make sure that I am in control of how I carry out my work |
|  | I make sure that I am in control of when I carry out my work |
|  | I make sure that I am in control of my work |
|  | I make sure that I am in control of the pace at which I carry out my work |
|  | I make sure that I am in control of my workload |
|  | I actively take on tasks that enable me to develop myself further |
|  | I consciously take on challenging tasks that give me energy |
|  | I apply for tasks that may be instructive to me |
|  | I actively try to build a good relationship with my supervisor |
|  | I ask my supervisor for support, if necessary |
|  | I ask my supervisor for his/her opinion about a problem concerning work, if necessary |
|  | I ask my co-workers for help, if necessary |
|  | I ask my co-workers to take over work from me, if necessary |
|  | I ask my co-workers for advice, if necessary |
|  | I ask my co-workers for their opinion about a problem concerning work, if necessary |
| Proactive burnout prevention in the home domain | |
|  | I make sure that I can decide for myself how to do things in my spare time |
|  | I make sure that I am in control of how I spend my free time |
|  | I make sure that I can organize my free time myself |
|  | I ask my family/friends for their opinion about a problem (concerning work), if necessary |
|  | I ask my family/friends for advice, if necessary |
|  | I ask my family/friends for help, if necessary |
|  | I make sure that I forget about work after hours |
|  | I make sure that I distance myself from work after hours |
|  | I make sure that I don’t think about work at all after hours |
| Proactive burnout prevention in the personal domain | |
|  | I try to view stressful situations from different angles |
|  | I try to put stressful situations into perspective |
|  | I stimulate a positive mindset in myself |
|  | I try to approach a problem positively |
|  | I make sure that I get enough exercise |
|  | I make sure that I engage enough in sports |
|  | I make sure I eat and drink healthy |
|  | I make sure that I do relaxing things after work |
|  | I makes sure that I kick back and relax after work |
|  | I make sure that I take time for relaxing activities after work |
